# Thiotrophic bacterial symbiont induces polyphenism in giant ciliate host *Zoothamnium niveum*

**DOI:** 10.1038/s41598-019-51511-3

**Published:** 2019-10-21

**Authors:** Monika Bright, Salvador Espada-Hinojosa, Jean-Marie Volland, Judith Drexel, Julia Kesting, Ingrid Kolar, Denny Morchner, Andrea Nussbaumer, Jörg Ott, Florian Scharhauser, Lukas Schuster, Helena Constance Zambalos, Hans Leo Nemeschkal

**Affiliations:** 1 University of Vienna, Department of Limnology and Bio-Oceanography, Vienna, Austria; 2 University of Vienna, Department of Theoretical Biology, Vienna, Austria

**Keywords:** Evolutionary ecology, Evolutionary developmental biology

## Abstract

Evolutionary theory predicts potential shifts between cooperative and uncooperative behaviour under fluctuating environmental conditions. This leads to unstable benefits to the partners and restricts the evolution of dependence. High dependence is usually found in those hosts in which vertically transmitted symbionts provide nutrients reliably. Here we study host dependence in the marine, giant colonial ciliate *Zoothamnium niveum* and its vertically transmitted, nutritional, thiotrophic symbiont from an unstable environment of degrading wood. Previously, we have shown that sulphidic conditions lead to high host fitness and oxic conditions to low fitness, but the fate of the symbiont has not been studied. We combine several experimental approaches to provide evidence for a sulphide-tolerant host with striking polyphenism involving two discrete morphs, a symbiotic and an aposymbiotic one. The two differ significantly in colony growth form and fitness. This polyphenism is triggered by chemical conditions and elicited by the symbiont’s presence on the dispersing swarmer. We provide evidence of a single aposymbiotic morph found in nature. We propose that despite a high fitness loss when aposymbiotic, the ciliate has retained a facultative life style and may use the option to live without its symbiont to overcome spatial and temporal shortage of sulphide in nature.

## Introduction

The notion that ‘all development is co-development’^1^ refers to the fact that hardly any animal or plant lives without microbial symbionts^2,3^. This entrenchment of environmental microbes^4^ has served innumerable times as an integral element for phenotypic construction and phenotypic novelty in eukaryote hosts^5–9^. Symbiont-induced developmental change in host traits has yielded complex phenotypes with adapted physiology to interact with the symbionts^3,8,10–13^.

The major transition from individuality of symbiotic partners in mutualistic relationships to a new integrated organism^14^ requires mutual dependence and alignment of partner interests^15,16^. Some symbionts are considered as being strictly required for normal host development and reproduction^9,10^, e.g. *Buchnera aphidicola –* aphids^3^, ‘*Candidatus* Endoriftia persephone’ – giant tubeworm^17^, ‘*Candidatus* Kentron’ – ciliate *Kentrophoros*^18,19^, *Polynucleobacter* – ciliate *Euplotes*^20–26^. Such obligate hosts bearing symbionts can display phenotypic plasticity – the ability of an organism to express different phenotypes in response to the varying environmental conditions^4,27–29^ - in various ways, but importantly, they do not retain the option to live aposymbiotically^30^.

In some other associations, however, not becoming irreversibly dependent has major evolutionary advantages. As environmental conditions strongly impact the outcome of interactions^2,3^, the elimination of aposymbiotic hosts is not selected for. Such facultative hosts have the option to interact with the partner as well as to live aposymbiotically^1,8^. For example, legumes develop root nodules in response to rhizobia infection only when fields are not fertilized with nitrogen^31^. The symbiotic and aposymbiotic anemone *Anthopleura elegantissima* occurs, according to light regime, either with *Symbiodinum* on sun-exposed or without these microalgae on shady substrates^32^. The endosymbiotic R-body producer *Caedibacter* transforms the host *Paramecium* into a killer of other paramecia including sometimes aposymbiotic individuals of the same species^33,34^. Most often *Paramecium bursaria* is found with photoautotrophic *Chlorella variabilis*^35^ but also natural aposymbiotic hosts exist^36^.

Some hosts, however, are never found in nature without their symbionts although they can be experimentally purged from them and still grow and sometimes even reproduce, e.g. *Aliivibrio fischeri* and bobtail squids^37^, methanogen bacteria and *Metopus contortus* ciliate^38^. To estimate host dependence over a range of animal and plant associations, the relative drop of host fitness between symbiotic and the experimentally purged or naturally occurring aposymbiotic hosts was calculated^39^. Overall hosts tend to be more dependent on vertically transmitted and nutritional symbionts than on horizontally transmitted and protective symbionts^39^. In ciliates fitness differences between symbiotic and aposymbiotic populations indicate the mutualistic advantage for the partners in nutritional^38^ and defensive associations^34^. Fitness, however, was found to be highly context dependent^40^. Under a variety of abiotic and biotic conditions ciliate mutualism might shift to a host exploiting the symbiont^41^ or a symbiont turning into a parasite^42^.

The mutualism between the vertically transmitted and nutritional, sulphur-oxidizing, chemoautotrophic (thiotrophic) ectosymbiont ‘*Candidatus* Thiobios zoothamnicoli’ and its giant colonial ciliate host *Zoothamnium niveum*^43,44^ is most suitable to test the hypothesis that symbiont function and transmission influence host dependence^39^. According to phylogenetic analyses based on the 18S rRNA gene sequence, *Z*. *niveum* belongs to the monophyletic clade 2^45^ (termed clade 1^46^) within a non-monophyletic genus^45–51^. Morphologically clade 2 species exhibit a colony growth pattern of alternate branches^45^. They share with all *Zoothamnium* species a suite of morphological characters, e.g. a common stalk connecting all zooids and containing a continuous spasmoneme that facilitates contraction in a “zigzag“ pattern^45,46,49^. Besides *Z*. *niveum*, most other clade 2 species are associated with ectosymbiotic bacteria: *Z*. *alternans*^52,53^, *Z*. *ignavum*^51^, Z. *pelagicum*^54–56^. Only in *Z*. *plumula* symbionts are not mentioned^57,58^.

In contrast to other *Zoothamnium* species of clade 2, which occur in oxic environments^51,53–55,57,59^, the *Z*. *niveum* partnership thrives in unstable and highly disturbed decaying plants and whale bones^60^ in the presence of hydrogen sulphide (∑H_2_S, i.e. sum of all forms of dissolved sulphide^61^, hereafter termed sulphide). The gammaproteobacterial symbiont has genes for sulphur oxidation and carbon fixation indicating a thiotrophic metabolism^62^.

Individual cells (termed zooids) of the colony are differentiated with different functions for division (terminal zooids), feeding (microzooids), and asexual reproduction (macrozooids). The terminal zooid on the tip of the stalk produces the terminal zooids on each branch, which in turn produce feeding microzooids and the macrozooids on the branches^63^. Vertical transmission of the ectosymbiont is through macrozooids that leave the colony as swarmers^43,44^, recruit to sulphide-emitting surfaces and grow into new colonies^64,65^. To date, the symbiont has neither been detected free-living in the environment nor has it been cultivated (MB unpublished data).

Through colony contraction and expansion, the colony dips into the sulphidic layer close to the substrate and extends into the oxic layer above, thereby alternately providing access to sulphide and oxygen as a byproduct benefit to the symbiont^60^. The symbiont fixes substantial amounts of inorganic carbon^66^. Host nourishment involves translocation of organic carbon to the host through both passive release, considered a byproduct benefit, and through digestion of symbionts^66^. Trading of goods in the mutualism, however, is interrupted when sulphide ceases during the cold season in temperate waters^67^.

The *Z*. *niveum* mutualism is currently the only thiotrophic association that can be cultivated over several generations^68^. Experiments of the host under oxic, sulphide supplemented, flow-through conditions lead to fast growth, long life span, and high reproduction. The colonies are white because they are covered by symbionts that store elemental sulphur. Under oxic, flow-through conditions host reproduction was reduced and colonies were pale and short-lived^68^. At that time we did not investigate whether these small colonies still carried symbionts. We therefore hypothesized that swarmers lose their symbionts during dispersal, that settlement is preferentially on sulphide-emitting surfaces but may also be possible on oxic surfaces, and that aposymbiotic swarmers grow into aposymbiotic colonies.

To test these hypotheses, we designed laboratory experiments under oxic conditions that mimic cessation of sulphide flux in nature to explore the response of swarmers and colonies, and the fate of the symbiont on the host. Here, we report on experimental evidence for a striking polyphenism, i.e., the development of discrete alternative phenotypes^30^. We show that the environmental conditions encountered by the swarmer lead either to loss or maintenance of the symbiont. This triggers the developmental switch, expressed in two distinct colony growth forms. We developed a growth form index for statistical comparison. Our experiments show that each growth form performed better under the respective environmental conditions. The 18S rRNA gene sequences of two colonies collected in the field – one resembling the aposymbiotically grown morph, the other the symbiotic morph – were identical. This confirmed that both morphs occur in nature.

## Results

### Swarmers during dispersal

**Figure 1:**
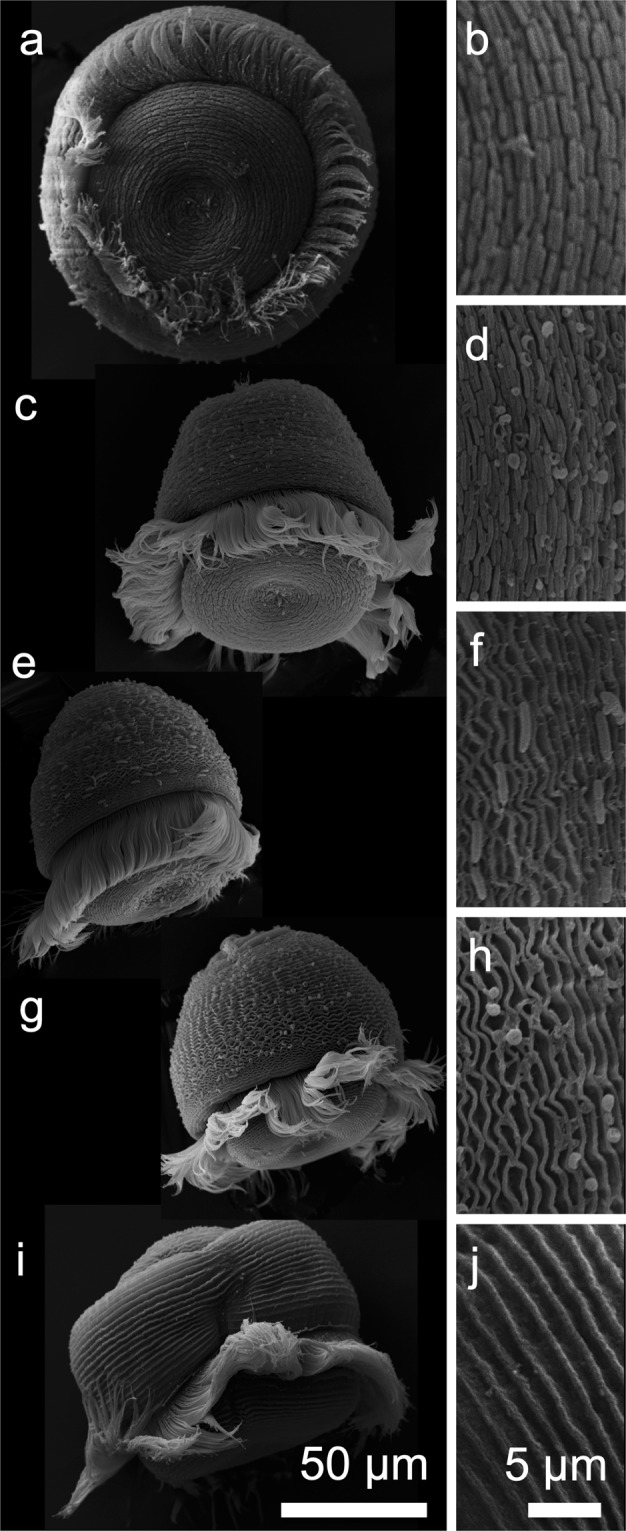
SEM micrographs of swarmers kept in oxic seawater for less than 4 h, and detail (**a**,**b**) between 4 h and 24 h old, fully covered with symbionts (**c**,**d**), partially covered with symbionts (**e**,**f**), with very few symbionts only, considered aposymbiotic (**g**,**h**) and between 24 h and 48 h old (**i**,**j**).

All swarmers kept under oxic, stagnant conditions for 4 h were fully covered by the symbiont, (n = 11, Fig. 1a,b and Supplementary Table S1). After 24 h, swarmers (n = 16) were either still fully covered (31%, Fig. 1c,d), partially covered (31%, Fig. 1e,f), or aposymbiotic (38%, Fig. 1g,h). All swarmers were aposymbiotic after 48 h (n = 14, Fig. 1i,j).

### Swarmer recruitment to sulphide-emitting surfaces

In oxic, stagnant seawater only about 1% of the released swarmers settled within 72 h (n ≈ 3,000). These swarmers were pale. Because of the low swarmer recruitment, we used a preference experiment (n = 6) to test whether reduced sulphur species (sulphide, thiosulphate) or low oxygen concentrations act as a settlement cue. Settlement checked after 24 h revealed an overall median of 22.5% recruited swarmers (interquartile range IQR 6.6%) exclusively to sulphide-emitting surfaces. Settlement was not significantly different between high and low sulphide concentrations (Wilcoxon-Mann-Whitney test, p-value 0.85, Supplementary Table S2). No settlement occurred at the thiosulphate, the reduced oxygen membranes, or the walls of the experimental chamber exposed to oxic seawater.

Because the swarmer recruitment experiment was carried out without chemical measurements, this experiment was repeated without swarmers to measure oxygen and sulphide concentration at the four membrane surfaces and revealed the presence of higher sulphide and lower oxygen at the high sulphide membrane and lower sulphide and higher oxygen at the low sulphide membrane until the end of the experiment. At the thiosulphate membrane, the oxygen concentration was similar to the concentration measured in the chamber, and sulphide was absent, whereas oxygen was very low and sulphide was absent at the membrane of the N_2_-bubbled vial (Supplementary Table S2).

To investigate whether this selective host behaviour was influenced by the presence of the symbiont on the host over time, a mix of aposymbiotic and symbiotic swarmers was exposed to sulphide under stagnant conditions to avoid removal of swarmers from the chamber under flow-through conditions (n = 17). A median of 47.7% (IQR 20.4%) swarmers recruited to surfaces within 23 h. Distance covariance tests showed that the time of sulphide exposure – between 2 and 23 h – had no influence on the number of settled symbiotic (p-value 0.28), aposymbiotic (p-value 0.27) and total swarmers (p-value 0.21) (Supplementary Table S3).

### Effects of sulphide and food supply on aposymbiotic host traits

**Figure 2:**
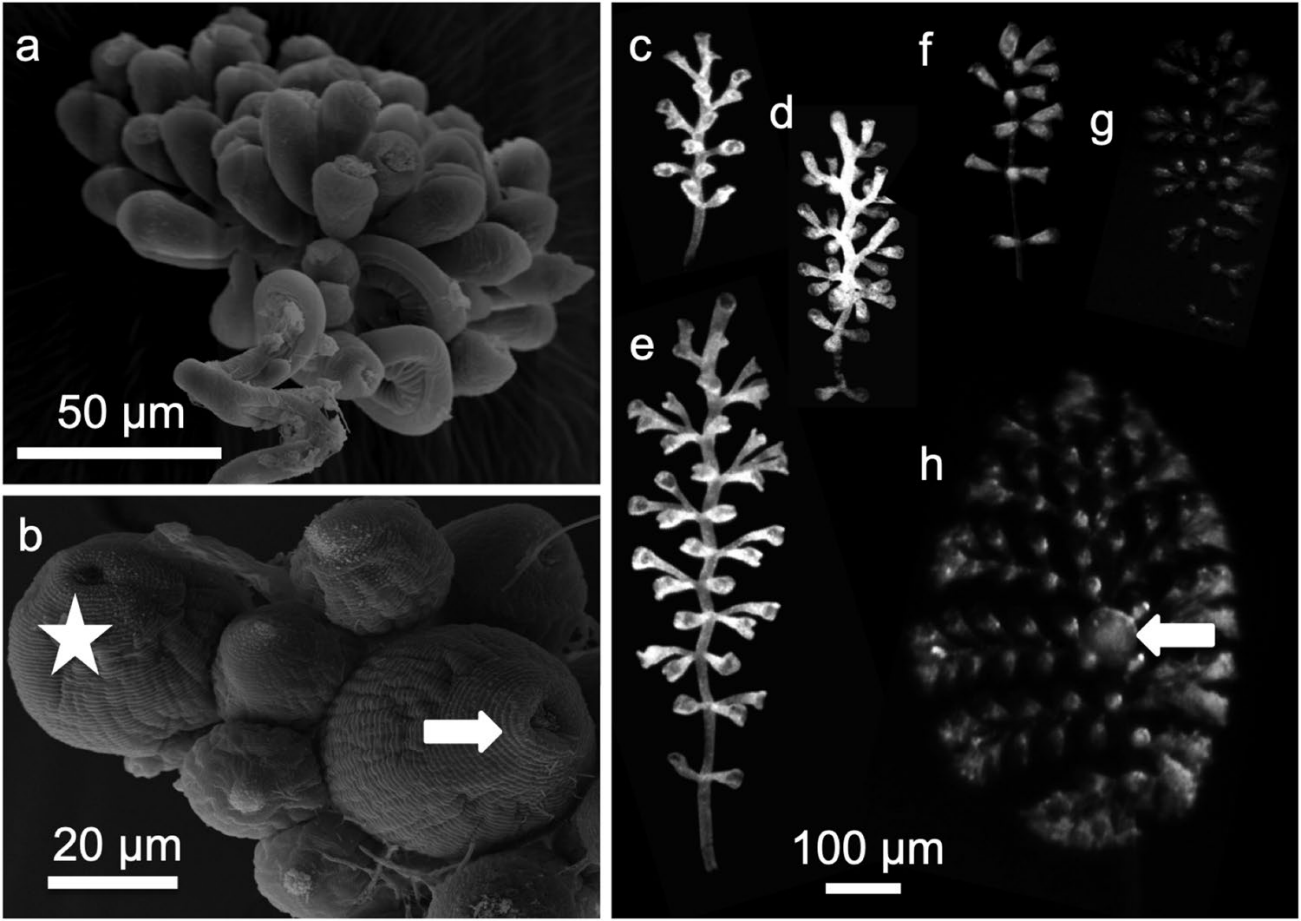
SEM micrographs of aposymbiotic colonies after seven days under sulphidic conditions (**a**), under oxic conditions with top terminal zooid (asterisk) and macrozooid (arrow) (**b**). Composite picture of light micrographs of symbiotic (**c**–**e**) and aposymbiotic morphs (**f**–**h**) macrozooid (arrow).

**Figure 3:**
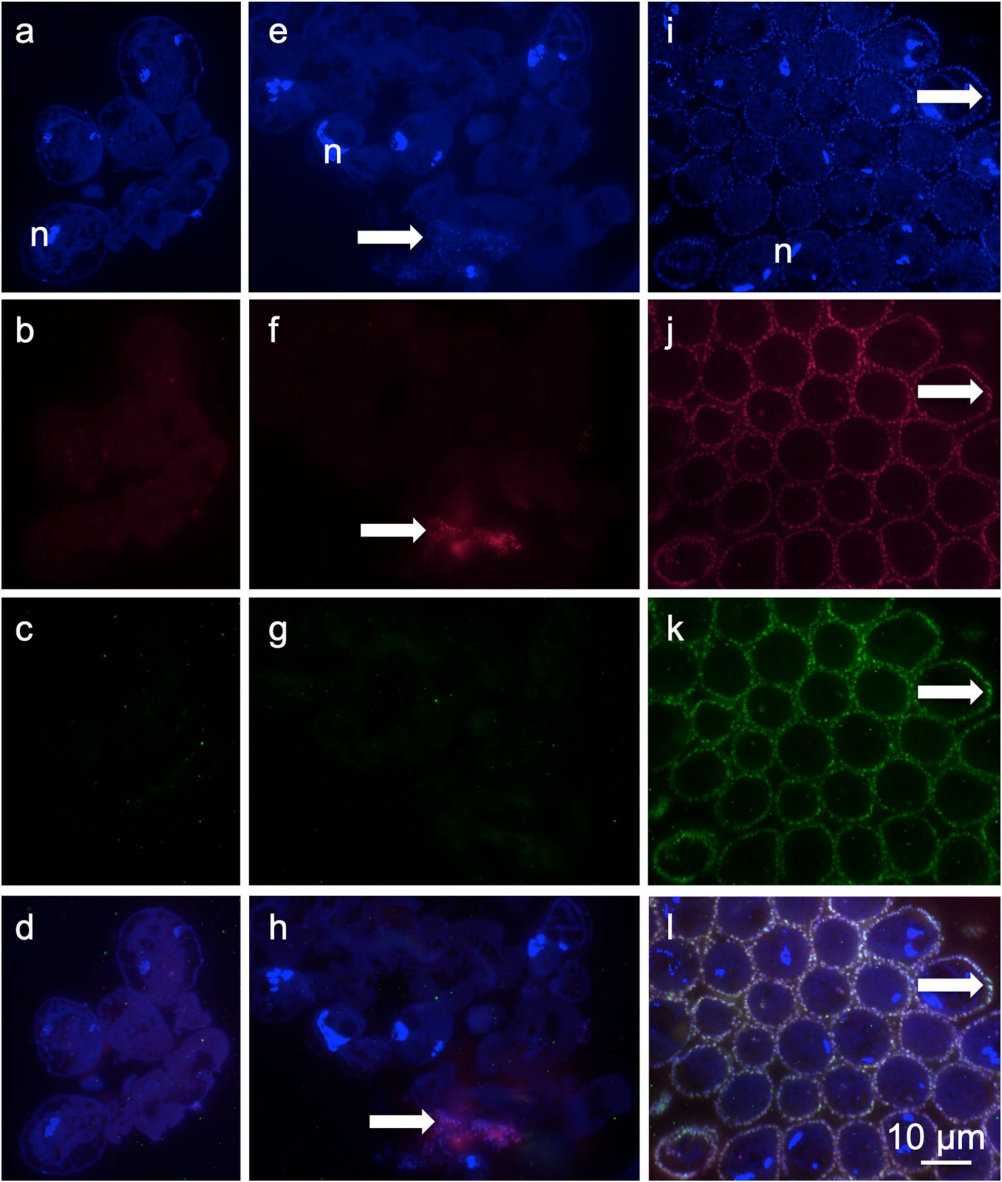
FISH micrographs of aposymbiotic morph grown under sulfidic conditions for seven days, stained with DAPI (**a**,**e**) (n macronuclei of host cells), labelled with EUB_mix_ and Arch915 probes (**b**,**f**), and symbiont-specific probe (**c**,**g**), and overlay (**d**,**h**) arrows points to microbes other than symbionts (**e**,**f**). Symbiotic morph grown under sulfidic conditions for seven days stained with DAPI (**i**)(n macronuclei of host cells), labelled with EUB_mix_ and Arch915 probes (**j**), and symbiont-specific probe (**k**) and overlay (**l**); arrows point to symbionts. Note that all micrographs are the same magnification.

Settled pale swarmers grew into colonies both under oxic, flow-through and oxic, sulphide-supplemented, flow-through conditions. At the end of the experiment at day 7 scanning electron micrographs (SEM) confirmed that most colonies were without microbial overgrowth (Fig. 2). Because in a few colonies some patches of microbes were seen, we performed fluorescence *in situ* hybridisation (FISH) on semithin sections using the symbiont-specific and the EUB_mix_ and Arch915 probes to identify the microbes. Also FISH micrographs revealed that most colonies were aposymbiotic (Fig. 3a–d). In some cases, however, other microbes colonized the host surfaces in small patches, which were labelled with the EUB_mix_ and Arch915 probes but not with the symbiont-specific probe (Fig. 3e–h). In contrast, the symbiotic, white colonies were covered with a monolayer of symbionts with positive EUB_mix_ and Arch915 and symbiont-specific labels (Fig. 3i–l). During the 7-day experiments no colony switched from pale to white under either chemical conditions. Under either condition, a few macrozooids were produced (Fig. 2b,h). Release of macrozooids and settlement of swarmers could not be followed in this experiment.

**Figure 4:**
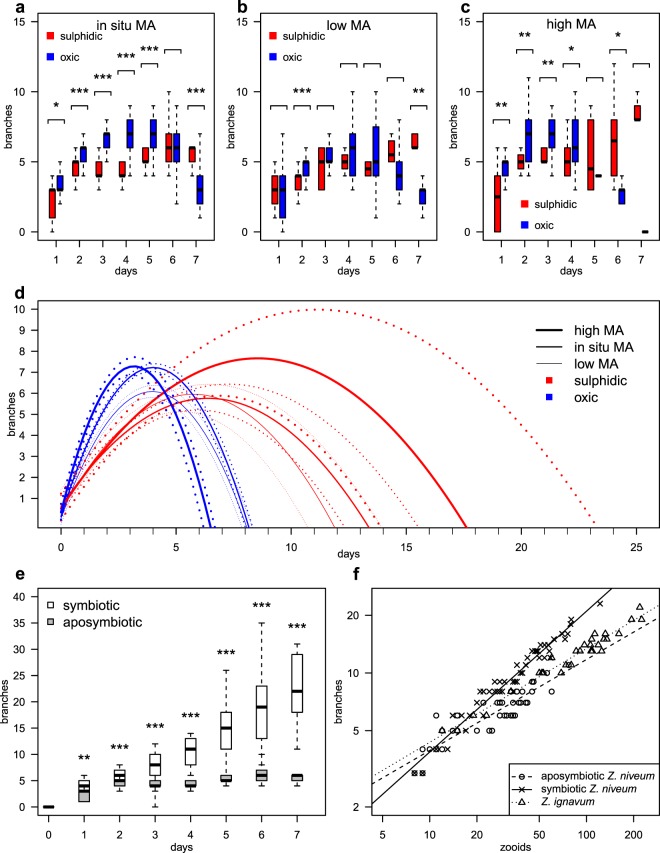
Box plots of growth (number of branches) of aposymbiotic colonies with *in situ* (**a**), low (**b**) and high (**c**) MA in sulphidic and oxic seawater (Wilcoxon-Mann-Whitney comparisons for each day; *p-value < 0.05, **p-value < 0.01, ***p-value < 0.001); (**d**) Estimated life span and maximal colony size of the aposymbiotic phenotype under sulphidic (red lines) and oxic (blue lines) conditions with reduced (thin line), *in situ* (medium line), and enhanced (thick line) MA, are inferred from the parabolas and 95% confidence intervals (dotted lines); (**e**) Box plots of growth of symbiotic and aposymbiotic colonies grown in the same chamber for seven days (Wilcoxon-Mann-Whitney comparisons for each day; **p-value < 0.01, ***p-value < 0.001); (**f**) Counted branches and total number of zooids in symbiotic and aposymbiotic colonies of *Z*. *niveum* and *Z*. *ignavum*, in double logarithmic scale of base 10 with power law fits.

At *in situ* microbial abundance (MA) aposymbiotic colonies had an estimated life span of 13.2 d (95% confidence interval 12.1, 15.3) under oxic, sulphide supplemented and 8.1 d (95% confidence interval 7.9, 8.2) under oxic conditions (Supplementary Table S4, Fig. 4a,d). The estimated maximal colony size reached a median of 7.2 branches (95% confidence interval 7.0, 7.4) in 4.0 d (95% confidence interval 4.0, 4.1) under oxic conditions and only 5.8 branches (95% confidence interval 5.2, 6.4) in 6.4 d (95% confidence interval 6.0, 7.3) under sulphidic conditions (Supplementary Table S4, Fig. 4a,d). Accordingly, colonies in oxic seawater grew faster with a relatively short, estimated life span, but to larger sizes, while oxic conditions supplemented with sulphide led to slow growth (hence longer life span) and smaller sizes.

Food density had no direct influence on the growth of aposymbiotic ciliates over a MA range of 2.0 × 10^5^ to 1.6 × 10^6^ mL^−1^. Under oxic conditions, however, reduced MA restricted maximal colony sizes versus the larger sizes under *in situ* and enhanced MA (Fig. 4a–d). Survival of aposymbiotic colonies under oxic conditions ranged from 0% (enhanced MA), 25% (reduced MA) to 30% (*in situ* MA) (Supplementary Table S4). Under oxic, sulphide supplemented conditions, reduced and *in situ* MA led to similar estimated maximal colony sizes, whereas enhanced MA resulted in larger sizes (Fig. 4a–d). Survival of aposymbiotic colonies under oxic, sulphide supplemented conditions ranged from 28% (reduced MA), 64% (enhanced MA) to 100% (*in situ* MA).

### Growth of symbiotic and aposymbiotic *Z*. *niveum* colonies

The morphology differed remarkably between colonies grown from aposymbiotic and symbiotic swarmers under oxic, sulphide supplemented conditions (Supplementary Table S5). Survival was 100% in aposymbiotic morphs and 66% in symbiotic morphs (Supplementary Table S5). After settlement, the small colonies were morphologically similar (Fig. 2c,d,f,g). During further development, however, the colonies with 10 (Fig. 2e,h) or 11 branches (Supplementary Fig. S2) differed between aposymbiotic and symbiotic phenotypes. This indicates that the proliferation activity of the terminal zooid of each branch was comparatively higher in the aposymbiotic than in the symbiotic phenotype. In contrast, the proliferation activity of the top terminal zooid was comparatively higher in the symbiotic compared to the aposymbiotic phenotype (Fig. 4e, Supplementary Table S5) leading to a long but narrow symbiotic morph and a short but wide, aposymbiotic morph. Aposymbiotic colonies grown from aposymbiotic swarmers under oxic conditions were identical in morphology to those grown under oxic, sulphide supplemented conditions (Fig. 2).

Comparing both morphs under oxic, sulphide supplemented, *in situ* MA conditions, growth was significantly higher in the symbiotic phenotype from day 2 to day 7 (Fig. 4e, Supplementary Fig. S1, Wilcoxon-Mann-Whitney tests, p < 0.001). At the end of the experiment on day 7, symbiotic colonies exhibited a median of 22 branches bearing an estimated 107 zooids, while aposymbiotic colonies had 6 branches with an estimated 24 zooids only (Supplementary Table S5). The aposymbiotic life span was limited to an estimated 13.2 d, but the symbiotic growth followed a monotonically increasing function (e.g. positive parabolic linear fit r^2^ 0.70, p-value 2 × 10^−44^, n = 169). The symbiotic morph therefore grew faster and to larger sizes. The estimated growth curve showed that the expected life span is much longer in the symbiotic than the aposymbiotic morph (Fig. 4e) confirming previous results^68^.

Under oxic, *in situ* MA conditions, none of the symbiotic morphs were found after seven days. Survival of aposymbiotic morphs was 30% (Supplementary Table S4). Recruited white swarmers turned pale within the first 24 h and grew into aposymbiotic morphs, which was confirmed with SEM at the end of the experiment at day 7.

### Growth form index (GFI)

A colony GFI (exponent of the power law relating zooids and branches) was obtained from aposymbiotic and symbiotic *Z*. *niveum*. Because the aposymbiotic morph highly resembled the symbiotic *Z*. *ignavum* in morphology we also obtained a GFI for comparison from this closely related species collected in the field^51^. The aposymbiotic morph had a GFI of 0.47 (n = 45, 95% confidence interval 0.40, 0.54), significantly different (p-value 5 × 10^−9^, analysis of covariance) from the symbiotic morph (GFI of 0.73, n = 51, 95% confidence interval 0.67, 0.78) (Fig. 4f, Supplementary Fig. S2). Symbiotic *Z*. *ignavum* showed a GFI of 0.49 (n = 30, 95% confidence interval 0.46, 0.52), non-significantly different to the aposymbiotic *Z*. *niveum* phenotype (p-value 0.73) but significantly different to the symbiotic *Z*. *niveum* phenotype (p-value 4 × 10^−12^) (Fig. 4f, Supplementary Fig. S2).

### Host 18S rRNA and symbiont 16S rRNA genes sequencing and phylogenetic analysis

**Figure 5:**
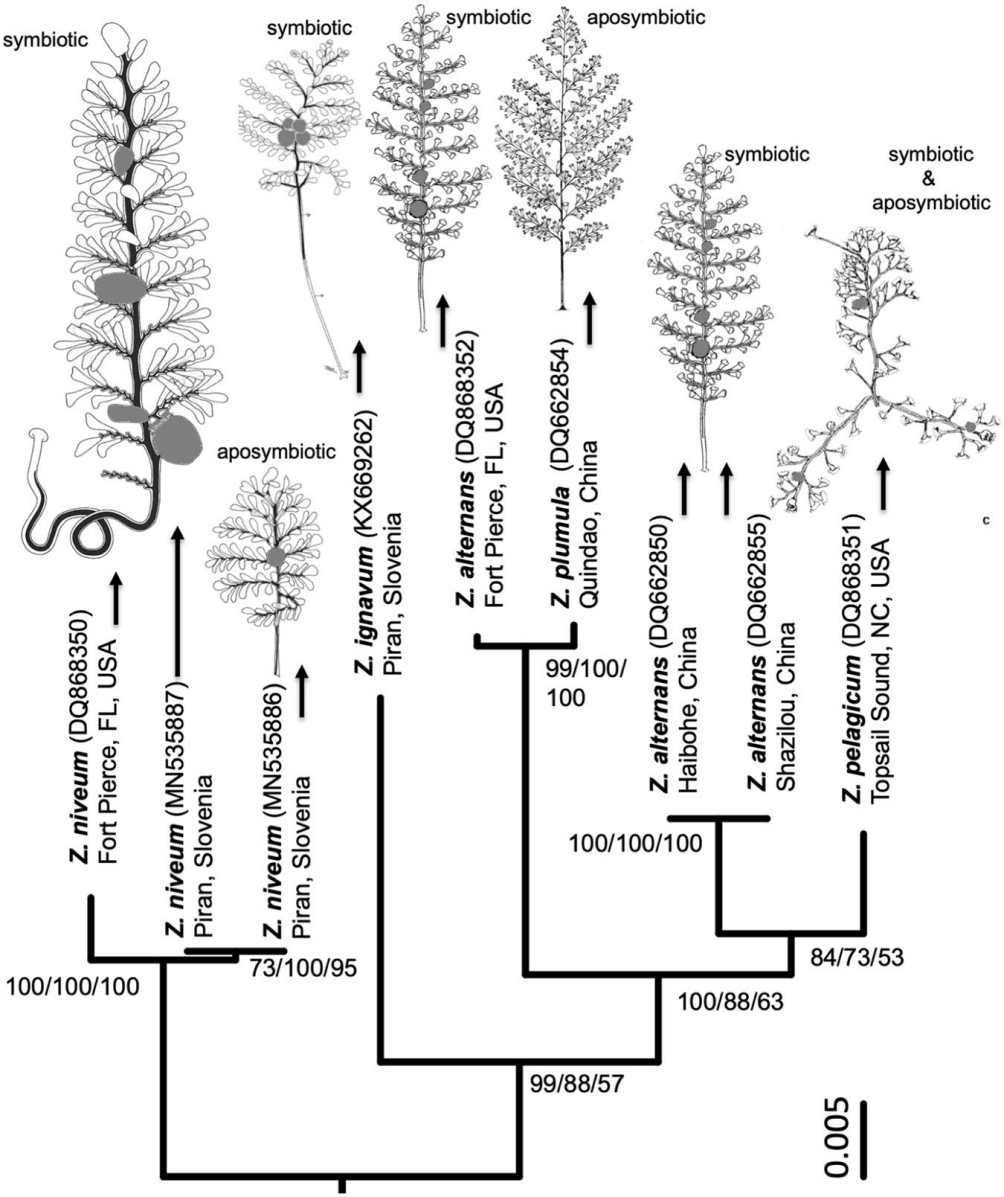
Bayesian tree inferred from the nucleotide sequences of the small subunit 18S rRNA gene of the monophyletic clade 2 of *Zoothamnium* and combined with colony drawings and life style. Support metrics are provided (BI/ML/MP). Scale bar corresponds to 1 substitution per 200 nt positions; numbers in parentheses are the NCBI accession numbers for each species. All colony drawings, including *Z*. *alternans*^53^, *Z*. *pelagicum*^54^, *Z*. *plumula*^57^, *Z*. *ignavum*^51^ and *Z*. *niveum* symbiotic^43^ and aposymbiotic *Z*. *niveum* (drawing from a colony grown in a flow-through chamber) show macrozooids in grey. Colony drawing of *Z*. *plumula*^57^ is under copyright and its use was granted by Magnolia Press www.mapress.com/j/zt. Colony drawings reproduction of *Z*. *alternans*^53^ was granted by the Instytut Biologii Doświadczalnej im. M. Nenckiego, and of *Z*. *niveum*^43^ by Elsevier. Colony drawing of *Z*. *pelagicum*^54^ is under copyright and its use was granted by CNRS Éditions (M. Laval; Zoothamnium pelagicum du Plessis. Cilié Péritriche planctonique: morphologie, croissance et comportement *in Protistologica n°4* ©CNRS Éditions, 1968).

Most of the colonies collected from minimally degraded wood and resembling the pale aposymbiotic phenotype were too low in DNA content to be sequenced (#4697/2-4,6,7). From one of these colonies (#4697/5, DNA content 3.6 ng/µL) 1,466 bp of the 18S rRNA gene could be retrieved. From a symbiotic, white colony collected from highly degraded wood (#4577, DNA content 32.4 ng/µL) 1,537 bp of the 18S rRNA gene were obtained. The sequence was identical to the pale colony (Supplementary Figs S3, S4) but differed to *Z*. *niveum* colony from USA^49^ (Sequence similarity 99.5%) (Supplementary Fig. S4). All three *Z*. *niveum* colonies build a monophyletic subclade in clade 2 supported by 100% posterior probability (Bayesian inference, BI) and 100% bootstrap support (Maximum Likelihood, ML; Maximum Parsimony, MP; Fig. 5). The presence of the symbiont 16S rRNA gene using general primers could only be obtained from the symbiotic colony indicating that the aposymbiotic phenotype was containing microbes (including the symbiont) too low in abundance to be sequenced (Supplementary Fig. S3).

## Discussion

**Figure 6:**
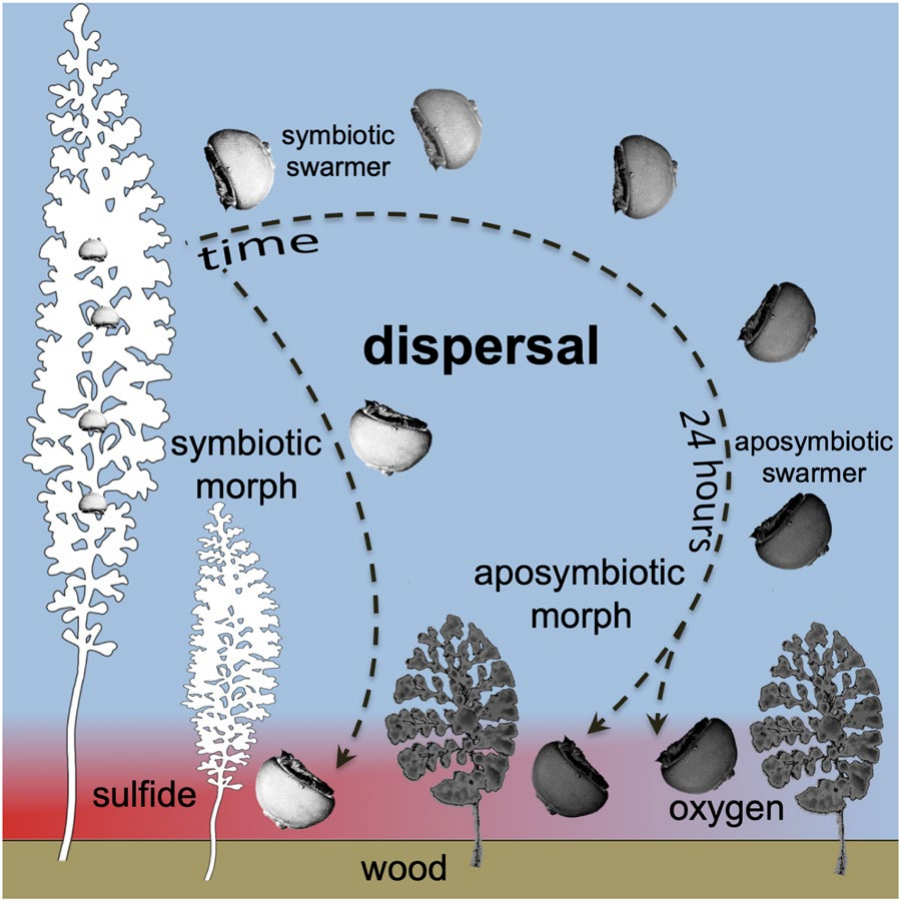
Proposed development of symbiotic morphs from symbiotic swarmers released from colony and short migration through oxic water prior to settlement on sulphide-emitting wood surface. In contrast, long migration of swarmers in oxic seawater leads to loss of symbionts and development of aposymbiotic morphs under both oxic and sulphidic conditions.

The thiotrophic mutualism between *Zoothamnium niveum* and its symbiont faces the challenge of maintaining long-term stability in a notoriously unstable environment. Sulphide and oxygen are not always available, and unless the host provides access to these chemicals to the symbiont, the symbiont cannot provide organic carbon to the host^41^. This study reports the discovery of one aposymbiotic ciliate in nature and shows experimentally, how this ciliate loses its symbiont when trading of goods between partners is interrupted under oxic conditions (Fig. 6). Most interestingly this ciliate exhibits a polyphenism in colony growth form. Despite high dependency, indicated by a fitness drop of 73% growth reduction in the aposymbiotic phenotype (Supplementary Table S5) and consistent with other hosts of vertically transmitted and nutritional symbionts^39^, this points to a facultative host. We propose that the aposymbiotic life style may ensure survival of the host at a temporal or spatial lack of sulphide in the environment.

New colonies can arise from swarmers under prolonged oxic conditions^68^. Here, we showed that swarmers easily lose the symbiont. Swarmers preferentially seek sulphidic surfaces for settlement to optimize symbiosis survival, but can also settle and grow aposymbiotically under unfavorable, oxic conditions, albeit to a very low percentage (Fig. 6). The time window for the swarmer to maintain its symbiont under oxic conditions is between 24 and 48 h. At a swarmer swim speed^69^ of 5 mm s^−1^, between 400 m and 800 m can be covered in one and two days, respectively. Accordingly, failing to find a sulphidic settlement near its release site leads to symbiont loss. The lack of a fully developed cytopharynx in swarmers suggests that symbionts cannot be digested^43^. It remains to be determined whether the host eliminates the symbiont because no benefits are provided and/or additional costs arise for the host to carry the symbiont during dispersal^70^, or whether the symbiont dies or leaves the host due to adverse conditions.

Our experiments indicate that sulphide is the settlement cue. This is consistent with previous experiments in which swarmers settled at the edge of sulphide point sources on cut-out blocks of mangrove peat placed in aquaria^65^. Only 1% of swarmers settled within 72 h during dispersal in oxic seawater. Although the percentage of recruited aposymbiotic colonies is low, in nature the high density of symbiotic populations producing large numbers of swarmers might nevertheless allow survival of aposymbiotic host populations. Based on an average number of 15.5 ± 2.8 (mean ± standard error) macrozooids per colony^63^ and 1,200 colonies m^−2^ on mangrove peat^64^) we estimate that well over 150 aposymbiotic colonies would be produced under oxic conditions from populations inhabiting a single square meter. Equally densely colonized wood (Supplementary Fig. S5), whale bones^71^, and seagrass debris^68^ have been documented elsewhere as well.

Most remarkably, this colonial ciliate exhibits a polyphenism in colony growth form, discernible as a long and narrow form in the symbiotic morph and a short and wide form in the aposymbiotic morph. The two morphs differ significantly in their growth form index. Polyphenism is well known from predator – prey, parasitic, and competitive interactions^72^. Other microbe – ciliate mutualisms are well known for their facultative host life style^73,74^. Often aposymbiotic and symbiotic morphs are quite similar in these unicellular hosts. *Metopus contortus* can be grown with its methanogen endosymbionts as well as aposymbiotically, revealing little morphological changes^38^. In the ciliate *Euplotidium itoi*, however, the presence of epixenosomes, unique ectosymbionts related to Verrucomicrobia^75^ defending their host against predators^76^, symbiotic morphs differ from artificially purged aposymbiotic morphs in the presence of a widened cortical region that the ectosymbionts are attached to^77^.

In the presence of sulphide in our experimental chamber, symbiotic and aposymbiotic swarmers gave rise to symbiotic and aposymbiotic morphs, respectively. In the absence of sulphide, only the aposymbiotic morph developed. In both cases the alternative phenotype exhibited significantly reduced growth indicating that each phenotype may be adaptive for the respective environment. The growth form expressed in oxic environments without symbionts experimentally would no doubt help the population to survive when swarmers fail to find a sulphidic substrate when such substrates are scarce or temporarily lacking at winter temperatures^67^. The finding of a single aposymbiotic morphotype of *Z*. *niveum* in nature may point to a much larger and more diverse habitat for this ciliate. Such aposymbiotic growth forms were not found on sulfide-emitting wood (MB pers. obs.). Whether host plasticity still retains the option to live aposymbiotically in nature or only in the lab by reverting to the growth form that they share with other species of clade 2 from oxic environments remains to be further investigated. The preferential recruitment behavior of the swarmer to abundant sulfide-emitting habitats and biological interactions such as competition and predation pressure may exclude the prevalence of aposymbiotic phenotypes when sulfide is produced during the warm seasons. They might be crucial, however, when temperature drops in late fall and ensure survival during the cold season.

The different growth of aposymbiotic morphs grown under oxic versus oxic, sulphide supplemented conditions suggests that sulphide stresses the aposymbiotic host and impacts growth. The mitochondria of invertebrates and protists from sulphidic environments detoxify sulphide to thiosulphate in the presence of oxygen^78,79^. Our experiments point to a trade-off between growth and longevity. Growth was fast and led to larger sizes but shorter life spans under oxic conditions. Under oxic, sulphide supplemented conditions the energy-consuming processes of sulphide detoxification may slow growth and extend the life span.

The aposymbiotic and symbiotic morphs of *Zoothamnium niveum* grown in experimental chambers differ remarkably in morphological traits used in the classification of species in this genus. Without experimental proof these two highly different morphs would be classified as different species. The 18S rRNA gene sequences from one colony collected from the field and resembling the aposymbotically grown morph in the lab as well as the 18S rRNA gene sequence from the symbiotic colony from the field were identical and allowed us to identify them as *Z*. *niveum*. Both colonies from the Adriatic Sea have 99.5% similarity in their 18S rRNA gene sequence with the sequence from USA^49^. In contrast, *Z*. *alternans* also from geographically distant locations and considered morphologically identical^53^ exhibit 96.7% sequence similarity^45^. This rather points to different species^51^ when applying a threshold between 97 to 99%^51^ or 99% sequence similarity^80^. Even more so, *Z*. *plumula* gene sequences from two relatively nearby locations in China^46,47^ fall in different clades^46^. Overall, the presence of cryptic species in the genus *Zoothamnium* and the discovered polyphenism in *Z*. *niveum* warrants to call for the addition of possible symbiont descriptions on morphological and molecular level as well as host gene sequences applied when new species are described and when population from previously unknown locations are studied.

The closely related species^49^ of *Zoothamnium niveum* in the clade 2 grow predominantly in oxic environments^51,53–55,57,59^. They show a wide range of either symbiotic or aposymbiotic life styles but share the diagnostic characteristic of a growth form with alternating branches^49^. They resemble, at first glance, the aposymbiotic morph with relatively long branches^51,53–55,57,59^ (Fig. 5). We could confirm the similarity of GFIs of aposymbiotic *Z*. *niveum* (0.47) and symbiotic *Z*. *ignavum* (0.49), but for other species data are not yet available. Visually *Z*. *alternans*^53^ highly resembles both aposymbiotic *Z*. *niveum* and *Z*. *ignavum*. Also relatively long branches are found in symbiotic *Z*. *pelagicum* that is in fact a “pseudo-colony“, composed of several ‘short and wide’ colonies growing on each other^54–56^ and in aposymbiotic Z. *plumula* with additional secondary branches^57^. Future research will help to determine whether specific growth forms can be related to specific environmental conditions.

## Conclusion

Our study revealed a mechanism of mutualism breakdown. Host development with and without the symbiont led to a polyphenism i.e. to discrete alternative colony growth forms triggered by the chemical conditions in the environment that the swarmer encounters. Prolonged oxic conditions lead to symbiont loss inducing the development of an aposymbiotic morph with reduced fitness, whereas symbionts were maintained during sulphidic or brief oxic conditions leading to the development of the symbiotic morph with high fitness. Whether aposymbiotic host populations play indeed a role in nature and if so, how the aposymbiotic host regains its symbiont to guarantee connectivity from aposymbiotic to symbiotic host populations remains to be studied.

## Methods

### Swarmers during dispersal

Colonies were collected from submerged wood at Sv. Jernej, Adriatic Sea, 2013 (replicate 1) and 2014 (replicate 2) (Supplementary Table S1). Colonies were cut from the substrate and transferred to embryo dishes filled with oxic seawater (24.5 °C ± 0.7 °C, salinity 33.3 ± 0.9, hereafter abiotic factors expressed as mean ± standard deviation). Release of swarmers was monitored after 4 h, 24 h, and 48 h (n = 2). Released swarmers were fixed for scanning electron microscopy (SEM) (Supplementary Table S1).

### Swarmer recruitment in preference chambers

Colonies were sampled from concrete blocks surrounded by seagrass debris in 2003 (Supplementary Table S2). For each experiment (n = 6), 30 to 50 released swarmers from about 50 colonies kept in oxic seawater (24 °C ± 1 °C, salinity 38 ± 1) were transferred into the central plastic cube (8 × 8 × 7 cm) of the preference chamber filled with oxic seawater. On each side of the cube, one vial was mounted and sealed with a dialysis polyethylene membrane (2.6 cm in diameter) permeable for small molecules to allow diffusion into the central chamber^81^ (Supplementary Fig. S1). The vials were filled with 30 mL seawater containing 1) ~1.5 mmol L^−1^ sulphide, 2) ~2.5 mmol L^−1^ sulphide, 3) 10 mmol L^−1^ thiosulphate, and 4) continuously N_2_-bubbled, anoxic seawater. After 24 h, the settlement of swarmers on the four membranes and on the inner cube’s surface was counted using a dissection microscope. Medians and quartiles were calculated and the nonparametric Wilcoxon-Mann-Whitney test was performed to assess differences between swarmer recruitment to high and low sulphide (R^82^ version 3.5.1, package *Coin*, v1.2-2).

Because abiotic parameters were not measured during this experiment in 2003, we repeated the experiment without swarmers to measure temperature, pH, salinity, oxygen, and sulphide in the chamber and oxygen and sulphide at all four membranes in October 2018 using a Fibox 4 (PreSenS, Germany) for oxygen and temperature, Multi 340i (WTW, Germany) for salinity and pH, and the Cline assay for ΣH_2_S^83^ (Supplementary Table S2).

### Swarmer recruitment in flow-through chambers

Colonies were collected from diverse submerged wood in the Adriatic Sea in 2013 and 2014 (Supplementary Table S3). Flow-through chambers^68^ (n = 17; Supplementary Fig. S1) were filled with 17 mL of oxic seawater containing sulphide (147 ± 35 µmol L^−1^). Between 90 and 227 pale, aposymbiotic and white, symbiotic swarmers were added to each chamber. The time for settlement was kept between 2 and 23 h during which the pumps were switched off to avoid swarmers being flushed out of the chamber. Afterwards, settled pale and white swarmers were counted, sulphide was measured, and flow through the chambers was established to follow colony growth (Supplementary Table S3). The covariance test (R package *Energy* v1.7-5) was used to show independence of time of sulphide exposure and number of settled swarmers.

### Effects of sulphide and food supply on aposymbiotic host traits

Colonies were collected from wood in Sv. Jernej in 2014 (Supplementary Table S4). For continuous flow through the chambers, we used an 8-channel peristaltic pump (Minipuls 3, Gilson International, Austria) set at 80 mL h^−1^ flow for oxic seawater and a syringe pump (KD Scientific, Inc., USA) set between 1.0 and 1.5 mL h^−1^ for sulphide supplementation in 50 mL syringes (between 1.5 and 1.8 mmol L^−1^ sulphide in Milli-Q water) (Supplementary Fig. S1). Three chambers were kept at oxic conditions, three others supplemented with sulphide. Two of these chambers (oxic and sulphidic) were fed with 32 µm filtered seawater to exclude eukaryote predation but to simulate the *in situ* microbial abundances (MA) commonly found in the northern Adriatic Sea during July^84^. To reduce the MA, we filtered the 32 µm pre-filtered seawater using the filter cartridge systems Polygard and Express SHC (Millipore, USA), with a final filtration of 0.2 µm pore size. To enhance the prokaryote density, we quadruple-concentrated 32 µm pre-filtered seawater using a Vivaflow 200 tangential flow module (Vivascience, Germany). Water and syringes were changed daily.

Abiotic parameters (flow, temperature, salinity, pH, oxygen, sulphide) and MA in the outflow water were monitored daily. Outflow water was filtered through polycarbonate filter membranes (Millipore GTBP02500 Isopore). Filters were fixed in 2% formalin for 24 h, air dried, and stored at −20 °C until staining with DAPI (4′,6-diamidino-2-phenylindole). MA was estimated by counting on an Axio Imager A1 epifluorescence microscope (Zeiss, Germany).

We followed survival as well as colony growth by counting the number of colonies and their respective branches under a dissection microscope daily. After 7 d the chambers were opened and colonies were fixed for SEM and fluorescence *in situ* hybridisation (FISH) (Supplementary Table S4). Shapiro-Wilk tests indicated deviations from normality for several of the experiments. Wilcoxon-Mann-Whitney tests were performed to assess differences of colony size between oxic and sulphidic conditions at low MA, *in situ* MA, and high MA. Linear regressions to quadratic polynomial parabolas (least squares fit) with Pearson R-square coefficients, and p-values were applied using R, because parabolas best reflected the growth and degenerative phase described for this ciliate^68^. Confidence intervals for parabolas were approximated by 10,000 random bootstrap re-samplings using the program ‘MUBOQB’.

### Growth of symbiotic and aposymbiotic *Z*. *niveum* colonies

Using colonies collected at Sv. Jernej in 2014, both white symbiotic and pale aposymbiotic settled swarmers (Supplementary Table S3, experiment 68) grew together into white symbiotic and pale aposymbiotic colonies, respectively, in one flow-through chamber under *in situ* MA and sulphidic conditions for seven days (Supplementary Table S4, experiment 68). Differences in colony size of aposymbiotic and symbiotic morphs after seven days were assessed using the Wilcoxon-Mann-Whitney test.

### Growth form index (GFI)

To estimate the relationship between cell population size (number of zooids) and colony size (number of branches), we selected micrographs of 96 *Z*. *niveum* (51 symbiotic and 45 aposymbiotic) from the above-described experiments and collections of white colonies at Sv. Jernej in 2014. For comparison we took photographs of 30 *Z*. *ignavum* colonies sampled at Sv. Jernej in 2014. A colony growth form index (GFI, exponent of the power law relating zooids and branches) was obtained from aposymbiotic and symbiotic *Z*. *niveum* and *Z*. *ignavum* as the slope of the log-log linear regression of the number of branches and zooids. This approach is similar to a study in which the topology of plant roots was characterized by comparing the ‘altitude’ (longest path length of root system, equivalent to number of branches) and the ‘magnitude’ (the number of root tips, equivalent to the number of zooids)^85^. The GFI describes the (log-transformed) number of branches that will develop from the addition of one (log-transformed) new zooid. The significance of the difference between the GFIs was assessed with a covariance analysis^86^ using R and by calculating of confidence intervals approximated by 10,000 random bootstrap re-samplings per analysis using ‘MUBOQB’.

### Scanning electron microscopy (SEM)

Colonies sampled at Sv. Jernej in 2014 (Supplementary Table S4) and swarmers collected in 2013 and 2014 (Supplementary Table S1) were fixed in Trump’s fixative (2.5% glutaraldehyde, 2% paraformaldehyde in 0.1 mol L^−1^ sodium cacodylate buffer 1100 mOsm L^−1^, pH 7.2) for up to 12 h, rinsed in 0.1 mol L^−1^ sodium cacodylate buffer, dehydrated in an ethanol series, transferred to 100% acetone, chemically dried with hexamethyldisilazane (EMS), coated with gold using a Sputter Coater 108 (Agar, United Kingdom), and observed on a IT 300 scanning electron microscope (JEOL, Tokyo, Japan). We distinguished symbiotic hosts with full coverage and partial coverage, and aposymbiotic hosts with less than 10 symbionts in 1,000 µm^2^.

### Fluorescence *in situ* hybridisation (FISH)

Colonies sampled at Sv. Jernej in 2014 (Supplementary Table S4) were fixed in 100% ethanol, embedded in the medium grade LR White resin, polymerized under nitrogen atmosphere at 40 °C for three days, and several semi-thin (0.5 µm) sections were placed in four drops of MilliQ water on gelatin-coated slides and air dried. Hybridisation was carried out according to Volland *et al*.^66^ using the symbiont-specific oligonucleotide probe ZNS196_mod labelled with Cy3 and the EUB_mix_ probes EUB338-I, EUB338-II, EUB338-III^87^ together with the Arch915 probe^88^ labelled with Cy5 to target most bacteria and archaea on sections placed into two drops. The sections of the other two drops of each slide were used for the nonsense probes labelled in Cy3 and Cy5 to control for false positives and no signals were observed.

### Host 18S rRNA and symbiont 16S rRNA genes sequencing and phylogenetic analysis

Colonies resembling the pale, aposymbiotic growth form of *Z*. *niveum* (#4697/2-7) were collected from minimally degraded, most likely oxic parts of wood in July 2018. For comparison, also white, symbiotic *Z*. *niveum* (#4577) was collected nearby but from highly degraded, most likely sulphide emitting parts of wood. Samples were fixed in 100% ethanol, and DNA was extracted with DNeasy blood tissue kit (Quiagen, Germany). DNA content was measured with a Nanodrop 2000 spectrophotometer. The primers 82F^89^ and MedlinB^90^ were used for PCR amplification of the 18S rRNA gene. Gel electrophoresis was performed on a 1% Agarose gel in TBE buffer for 50 minutes at 90 Volt. For the 16S rRNA gene the primers 27F^91^ and 1492R^92^ were used. Bidirectional Sanger sequencing was performed (Microsynth AG, Switzerland). Sequences were analyzed with Geneious v. 11.1. (Biomatters, New Zealand).

To test the close affiliation between *Z*. *niveum* from Fort Pierce, USA and *Z*. *niveum* from Piran, Slovenia the nucleotide sequences of the small subunit rRNA genes of all members of clade 2^46,51^ were obtained from NCBI, aligned with MAFFT^93^ v7.407 and trimmed to the shortest sequence length obtained from the collected pale phenotype (1,466 bp) using SeaView^94^ version 4 and ape^95^ v5.2 package of R: *Z*. *niveum* DQ868350, Fort Pierce, FL, USA^49^; *Z*. *alternans* DQ868352, Fort Pierce, FL USA^49^; *Z*. *alternans* DQ662855, Shazikou, China^47^; *Z*. *alternans* DQ662850, Haibohe, China^47^; *Z*. *pelagicum* DQ868351 Topsail Sound, NC, USA^49^; *Z*. *plumula* DQ662854, Quindao, China (published under the name *Z*. *pluma*)^47^; *Z*. *ignavum*. KX669262, Piran, Slovenia^51^. *Z*. *plumula* KY675162, Yantai, China^46^ was used as outgroup. The 18S rRNA eukaryote gene sequences of both collected colonies were deposited in the GenBank database under accession number MN535886 (Aposymbiotic_*Zoothamnium_niveum*_str._Piran_4697/5) and MN535887 (Symbiotic_*Zoothamnium_niveum*_str._Piran_4577).

Maximum Likelihood and Maximum Parsimony phylogenies with bootstrap support were calculated with ape and phangorn^96^ v2.4.0 packages in R, under a GTR + I nucleotide substitution model (with best corrected AIC value). Bayesesian inference phylogeny with posterior probability support was generated with MrBayes^97^ v3.2.7a with 1,750,000 generations and a burn-in of 25% of the length. The obtained tree was combined with colony drawings of a symbiotic colony^43^ and a aposymbiotically grown colony of *Z*. *niveum* (this publication), *Z*. *alternans*^53^, *Z*. *pelagicum*^54^, *Z*. *plumula*^57^, and *Z*. *ignavum*^51^ and data on life style^43,51,53–55,57,59^. Reproduction of *Z*. *plumula*^57^ drawing was granted by Magnolia Press, of *Z*. *alternans*^53^ by the Instytut Biologii Doświadczalnej im. M. Nenckiego, of *Z*. *niveum*^43^ by Elsevier, and *Z*. *pelagicum*^54^ by CNRS Éditions.

## Supplementary information

Supplementary Information

## Data Availability

The datasets generated during and/or analysed during the current study are included in this published article and its Supplementary Information Files and are available in the FigShare repository, https://figshare.com/s/98e63972a493c272930e.
