# Supplementary material for: Thiotrophic bacterial symbiont induces polyphenism in giant ciliate host Zoothamnium niveum
Source: Sci Rep. 2019 Oct 21;9:15081. doi: 10.1038/s41598-019-51511-3 (PMC6803713; doi:10.1038/s41598-019-51511-3)
Supplement: Supplementary file 1 — Supplementary Information [file 41598_2019_51511_MOESM1_ESM.docx]

**Supplementary Information**

**Thiotrophic bacterial symbiont induces polyphenism in giant ciliate host *Zoothamnium niveum***

Monika Bright^1*^, Salvador Espada-Hinojosa^1^, Jean-Marie Volland^1^, Judith Drexel^1^, Julia Kesting^1^, Ingrid Kolar^1^, Denny Morchner^1^, Andrea Nussbaumer^1^, Jörg Ott^1^, Florian Scharhauser^1^, Lukas Schuster^1^, Helena Constance Zambalos^1^, Hans Leo Nemeschkal^2^

^1^University of Vienna, Department of Limnology and Bio-Oceanography, Vienna, Austria

^2^University of Vienna, Department of Theoretical Biology, Vienna, Austria

(*) Correspondence:

M. Bright, University of Vienna, Department of Limnology and Bio-Oceanography, Althanstr. 14, A-1090 Vienna, Austria.

Phone: +43 1 4277 76430; Fax: +43 1 4277 876401; Email: [monika.bright@univie.ac.at](mailto:Monika.bright@univie.ac.at)

**Supplementary Figure S1**

(**a**) Hard PVC preference chamber with central cube (cc) and four attached vials with gas-permeable membranes (arrowheads) to allow gas diffusion and swarmer settlement. Two cylinders were filled with low (ls) and high (hs) sulphide concentrations, the third cylinder with thiosulphate (ts) solution, and the fourth cylinder with anoxic seawater (as). (**b**) Flow-through system with 8-channel peristaltic pump providing constant seawater flow to the plexiglas chamber through the upper inlet, and syringe pump providing sulfide to the chamber through the lower inlet. (**c**) Chamber with upper and lower inlet and outlet. (**d**) Close-up at the upper corner of the chamber showing both white symbiotic colonies and pale aposymbiotic colonies (arrows) growing next to each other.

**
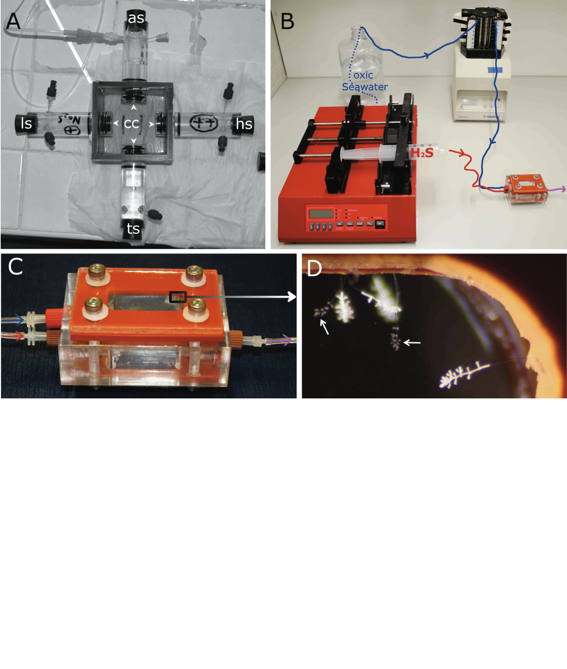
**

**Supplementary Figure S2**

Illustration of growth form of symbiotic and aposymbiotic *Z. niveum* and symbiotic *Z. ignavum*. Note that all three colonies exhibit 11 branches, but differ the number of zooids on each branch. First number = branch number, second number = number of zooids on this branch; each ending delineates a zooid, which is not drawn.

symbiotic *Z. niveum* aposymbiotic *Z. niveum* symbiotic *Z. ignavum*

**
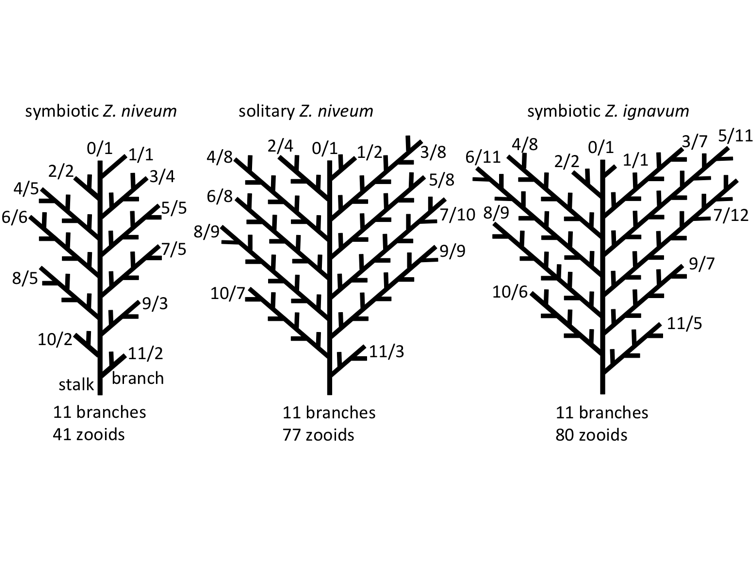
**

**Supplementary Figure S3**

Gel electrophoresis pictures showing PCR products of 18S (**a**) and 16S (**b**) rRNA genes (SZ = size marker = Gene ruler^TM^ 1kb DNA Ladder, Waltham, Massachusetts, USA; colonies collected in the field resembled aposymbiotically grown morph (# 4697/2-5): 2-5h = host, 2-5s = symbiont; pos = positive control of colony (# 4577) collected in field resembled symbiotically grown morph; neg = negative control).

**
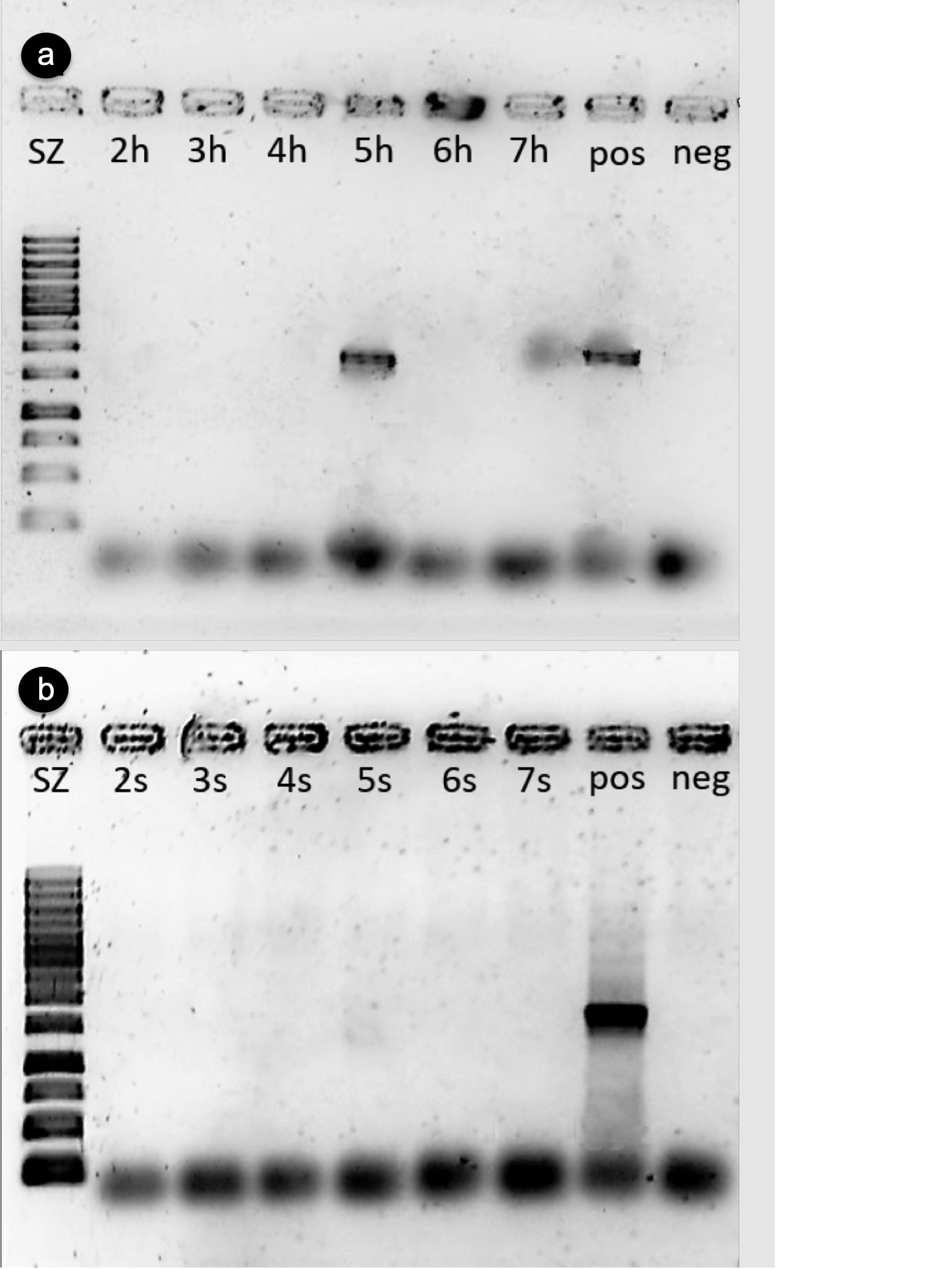
**

**Supplementary Figure S4**

Alignment of 18S rRNA gene from *Zoothamnium niveum*: aposymbiotic morph (# 4697/5, NCBI accession number MN535886) and symbiotic morph (# 4577, NCBI accession number MN535887) from Piran, Slovenia and *Z. niveum* (DQ868350.1) from Fort Pierce, FL, USA.

**
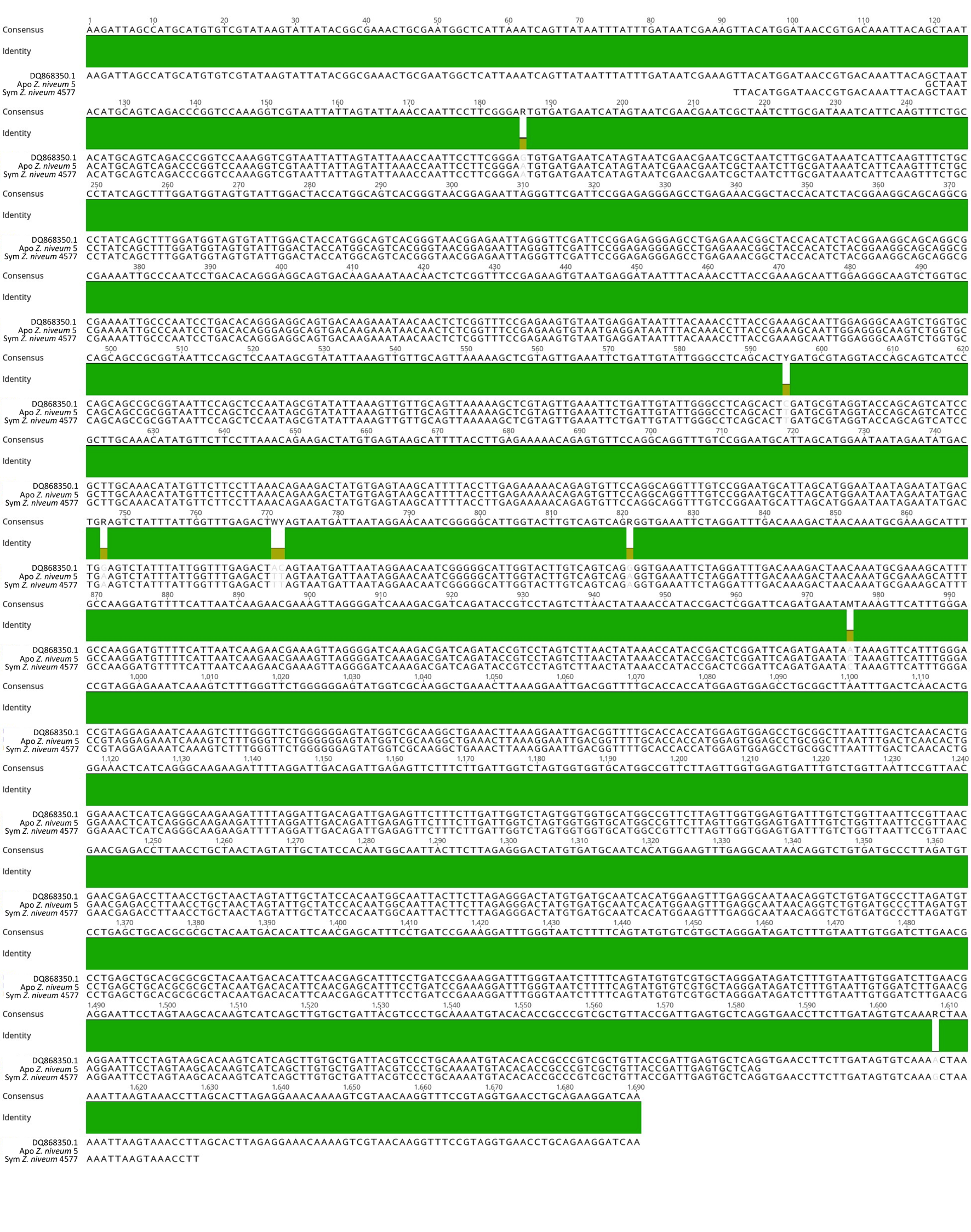
**

**Supplementary Figure S5**

Micrograph of colonies grown on submerged wood.

**
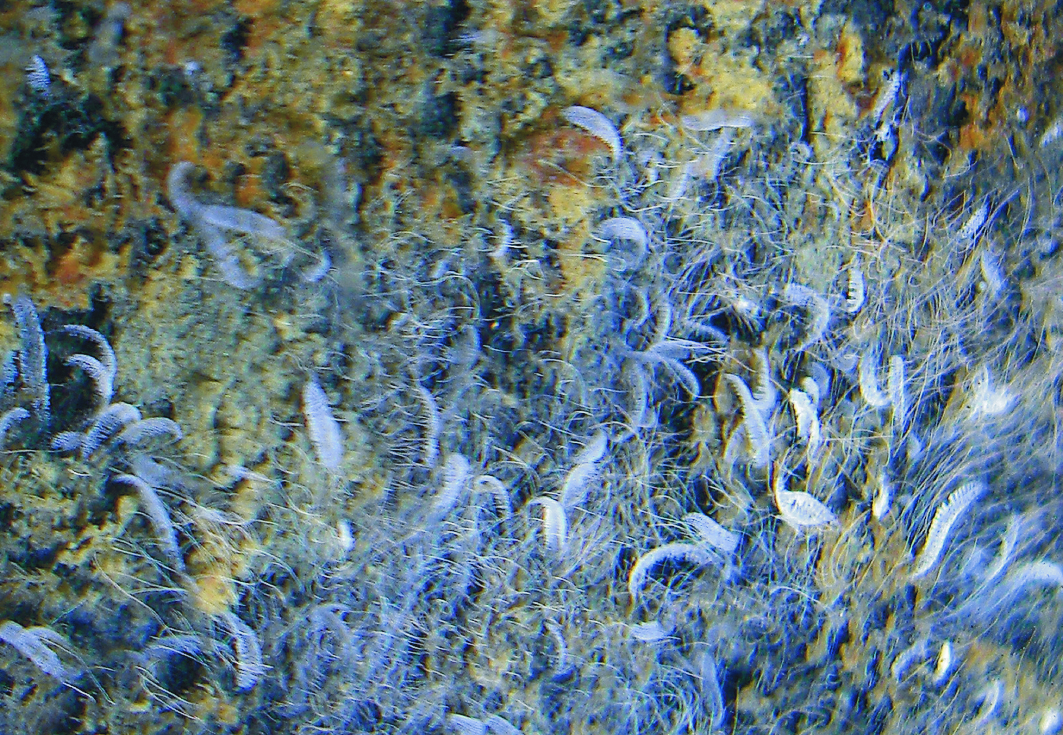
**

**Supplementary Table S1: Swarmers during dispersal**

Replicate 1 and 2 with wood number from with colonies were retrieved, sampling location with latitude and longitude, depth, and sampling date; number of colonies used for producing swarmers in embryo dishes, number of initial swarmers that were incubated under oxic conditions between 4 and 48 h and analyzed with SEM.

| **Replicates** | **1** | **2** |
| --- | --- | --- |
| Sampling wood number | 41 | 61 -63 |
| Sampling location | Sv. Jernej | Sv. Jernej |
| Latitude | 45.498286 N | 45.498286 N |
| Longitude | 13.59352 E | 13.593521E |
| Depth (m) | 0.5 | 0.5 - 1.5 |
| Sampling date | 7 July 2013 | 9 July 2014 |
| Colonies used for swarmer release | 10 | 120 |
| Number of initial swarmers | 15 | 240 |
| **Incubation time (h)** | **Number of swarmers analysed with SEM** | |
| 4 | 3 | 8 |
| 24 | 6 | 10 |
| 48 | 4 | 10 |

**Supplementary Table S2: Swarmer recruitment to sulfide in preference chambers**

(**a**) Sampling location, latitude, longitude, depth, and sampling date of colonies from two concrete blocks surrounded by seagrass *Posidonia oceanica* debris used for producing swarmers in embryo dishes under oxic conditions for settlement preference experiment with six replicates, showing number of colonies used for swarmer release, initial number of swarmers and settlement of swarmers exclusively colonizing membranes emitting high and low sulphide within 24 hours.

| **Sampling** |  |  |  |  |  |  |
| --- | --- | --- | --- | --- | --- | --- |
| Sampling location | concrete blocks, Bay of Stareso, Corse | | | | | |
| Latitude | 42.58047 N | | | | | |
| Longitude | 8.72438 E | | | | | |
| Depth | 7 - 10 m | | | | | |
| Sampling date | September 2003 | | | | | |
| Colonies used for swarmer release | 50 | | | | | |
| **Replicates** | **1** | **2** | **3** | **4** | **5** | **6** |
| Number of initial swarmers | 40 | 40 | 50 | 50 | 30 | 30 |
| Number of settled swarmers |  |  |  |  |  |  |
| Low sulphide | 5 | 5 | 3 | 8 | 1 | 4 |
| High sulphide | 3 | 5 | 1 | 2 | 8 | 4 |

(**b**) The experiment was repeated without swarmers in October 2018 to measure the abiotic conditions (sulphide, oxygen, temperature, salinity, pH) in the chamber at the beginning (0h) and after 24h; sulfide and oxygen concentrations were measured at the four membranes facing the chamber after 1h, 5 h, 18 h, and 24 h (mean ± standard deviation).

| **Replicate chemistry** | **time** | **ΣH_2_S (µmol L^-1^)** | **O_2_ (µmol L^-1^)** | **T (°C)** | **Salinity** | **pH** |
| --- | --- | --- | --- | --- | --- | --- |
| In chamber | 0 h | 0 | 211 | 19.4 | 32 | 8.2 |
|  | 24 h | 0 | 167 | 19.1 | 33 | 8.3 |
| Low sulphide | 1 - 24 h | 112 ± 45 | 54 ± 18 |  |  |  |
| High sulphide | 1 - 24 h | 386 ± 25 | 23 ± 11 |  |  |  |
| Thiosulphate | 1 - 24 h | 0 | 186 ± 20 |  |  |  |
| N_2_ - bubbled | 1 - 24 h | 0 | < 1 |  |  |  |

**Supplementary Table S3: Swarmer recruitment in flow-through chambers**

Wood number from which colonies were sampled, Sampling location with latitude and longitude, depth, and sampling date of colonies from submerged wood used for producing swarmers in embryo dishes incubated under oxic conditions and transferred to flow-through chambers for recruitment experiment under oxic condition between 2 and 22 hours; 17 replicates with replicate number provided; for each replicate time of settlement of swarmers, initial number of swarmers, total number of settled swarmer, and number of settled symbiotic and aposymbiotic swarmers are provided; aposymbiotic and symbiotic swarmers were counted after settlement.

| **Sampling wood number** | **Sampling location** | **Latitude** | **Longitude** | | **Depth**  **(m)** | | **Sampling date** | **Replicate number** | | **Time of settle-ment (h)** | **Number of initial swarmers** | | | | **Total number of settled swarmers** | **Number of symbiotic settled swarmers** | | | | **Number of apo-symbiotic settled swarmers** |
| --- | --- | --- | --- | --- | --- | --- | --- | --- | --- | --- | --- | --- | --- | --- | --- | --- | --- | --- | --- | --- |
| 59 | Sv. Jernej | 45.49829 N | 13.59352 E | | 1.5 | | 4 July 2014 | 70 | | **2** | 120 | | | | 46 | 11 | | | | 35 |
| 59 | Sv. Jernej | 45.49829 N | 13.59352 E | | 1.5 | | 4 July 2014 | 71 | | **2.5** | 120 | | | | 48 | 45 | | | | 3 |
| 41 | Sv. Jernej | 45.49829 N | 13.59352 E | | 0.5 | | 7 July 2013 | 43 | | **3.5** | 227 | | | | 90 | 35 | | | | 55 |
| 61 -63 | Sv. Jernej | 45.49829 N | 13.59352 E | | 0.7 | | 9 July 2014 | 77 | | **7.5** | 120 | | | | 75 | 59 | | | | 16 |
| 70 | Sv. Jernej | 45.49829 N | 13.59352 E | | 1.0 | | 16 July 2014 | 79 | | **7.5** | 150 | | | | 79 | 67 | | | | 21 |
| 70 | Sv. Jernej | 45.49829 N | 13.59352 E | | 1.0 | | 16 July 2014 | 78 | | **9** | 120 | | | | 61 | 48 | | | | 13 |
| 72-76 | Strunjan | 45.52810 N | 13.60406 E | | 0.7 - 1.5 | | 22 July 2014 | 84 | | **9** | 90 | | | | 59 | 50 | | | | 9 |
| 72-76 | Strunjan | 45.52810 N | 13.60406 E | | 0.7 - 1.5 | | 22 July 2014 | 86 | | **9.5** | 90 | | | | 26 | 9 | | | | 17 |
| 72-76 | Strunjan | 45.52810 N | 13.60406 E | | 0.7 - 1.5 | | 22 July 2014 | 87 | | **9.5** | 90 | | | | 36 | 28 | | | | 8 |
| 72-76 | Strunjan | 45.52810 N | 13.60406 E | | 0.7 - 1.5 | | 22 July 2014 | 88 | | **9.5** | 150 | | | | 40 | 14 | | | | 26 |
| 60 - 63 | Sv. Jernej | 45.49829 N | 13.59352 E | | 0.3 - 0.7 | | 9 July 2014 | 74 | | **11** | 160 | | | | 51 | 34 | | | | 17 |
| 60 - 63 | Sv. Jernej | 45.49829 N | 13.59352 E | | 0.3 - 0.7 | | 9 July 2014 | 75 | | **12.3** | 200 | | | | 97 | 61 | | | | 36 |
| 70 | Sv. Jernej | 45.49829 N | 13.59352 E | | 1.0 | | 16 July 2014 | 83 | | **12.5** | 105 | | | | 35 | 10 | | | 25 | |
| 56 - 58 | Sv. Jernej | 45.49829 N | 13.59352 E | | 0.5 - 1.0 | | 29 June 2014 | 68 | | **13** | | | 132 | 129 | | | 91 | | 38 | |
| 41 | Sv. Jernej | 45.49829 N | 13.59352 E | 0.5 | | 7 July 2013 | | 39 | **21** | | | 220 | | 105 | | | 80 | 25 | | |
| 41 | Sv. Jernej | 45.49829 N | 13.59352 E | 0.5 | | 7 July 2013 | | 41 | **23** | | | 218 | | 158 | | | 144 | 14 | | |
| 41 | Sv. Jernej | 45.49829 N | 13.59352 E | 0.5 | | 7 July 2013 | | 42 | **22** | | | 218 | | 105 | | | 80 | 25 | | |

**Supplementary Table S4.** **Effects of sulphide and food supply on aposymbiotic host traits**

Sampling location and date of collection of colonies from submerged wood (wood number and depth) used for producing swarmers in embryo dishes used as inoculum (number of total swarmers, number of settled aposymbiotic swarmers, time of settlement); experiments under environmental conditions (ΣH_2_S and O_2_ concentration, temperature, salinity, pH, water flow through chambers, microbial abundance in seawater shown as median and Q_25_ and Q_75_) used for growing aposymbiotic colonies for seven days; colony size as number of branches, number of colonies, colony survival at day 7 are counted; life span, maximal colony size and time when maximal colony size is reached are estimated; numbers of colonies analyzed with scanning electron microscopy (SEM) and fluorescent in situ hybridisation (FISH) at the end of the experiment are shown.

| **Experiment** | **77** | **75** | **68** | **72** | **78** | **83** |
| --- | --- | --- | --- | --- | --- | --- |
| **Sampling** |  |  |  |  |  |  |
| Wood number | 61 - 63 | 61 - 63 | 56 - 58 | 59 | 70 | 70 |
| Sampling location Sv. Jernej (date) | 9 July 2014 | 9 July 2014 | 29 June 2014 | 4 July 2014 | 16 July 2014 | 16 July 2014 |
| Depth (m) | 0.7 | 0.7 | 0.5 - 1.0 | 1.5 m | 1.0 m | 1.0 m |
| **Swarmer inoculum** |  |  |  |  |  |  |
| Number of total swarmers | 120 | 200 | 132 | 120 | 120 | 105 |
| Number of settled aposymb. swarmers | 16 | 36 | 38 | N/A | 13 | 25 |
| Time of settlement (h) | 7.5 | 12.3 | 13.0 | 2.5 | 9.0 | 12.5 |
| **Experimental conditions** |  |  |  |  |  |  |
| ΣH_2_S (µmol L^-1^) | 21 ± 6 | 0 | 19 ± 7 | 0 | 24 ± 3 | 0 |
| O_2_ (µmol L^-1^) | 215 ± 7 | 221 ± 4 | 216 ± 5 | 216 ± 4 | 218 ± 4 | 212 ± 8 |
| Temperature (°C) | 25.3 ± 0.5 | 24.1 ± 0.4 | 25.1±0.6 | 25.0 ± 0.6 | 25.3 ± 0.3 | 25.5 ± 0.3 |
| Salinity | 32.9 ± 1.2 | 32.8 ± 0.7 | 31.8 ± 0.5 | 32.2 ± 0.7 | 33.1 ± 1.0 | 33.8 ± 1.2 |
| pH | 8.2 ± 0.0 | 8.1 ± 0.0 | 8.2 ± 0.0 | 8.1 ± 0.0 | 8.2 ± 0.0 | 8.2 ± 0.1 |
| Flow (mL h^-1^) | 84 ± 2 | 81 ± 4 | 63 ± 21 | 79 ± 6 | 82 ± 5 | 76 ± 13 |
| Microbial abundance (x10^5^ mL^-1^) | 2.0 (1.8, 2.9) | 3.0 (2.4, 3.8) | 7.9 (7.6, 8.0) | 7. 5 (7.3, 7.8) | 11.5 (9.3, 15.2) | 16.5 (15.6, 17.2) |
| **Colony growth and survival** |  |  |  |  |  |  |
| Colony size, day 7 (no. of branches) | 6 (6, 7) | 3 (2, 3) | 6 (6,7) | 3 (2, 4) | 8 (8, 9) | 0 |
| Number of colonies on day 7 | 5 | 25 | 21 | 30 | 9 | 0 |
| Colony survival on day 7 (%) | 28 | 25 | 100 | 30 | 64 | 0 |
| Estimated lifespan (d) | 12.3 | 8.0 | 13.2 | 8.1 | 17.4 | 6.4 |
| Estimated maximal colony size | 5.9 | 6.1 | 5.8 | 7.2 | 7.7 | 7.3 |
| Estimated time of maximal colony size (d) | 6.0 | 4.0 | 6.4 | 4.0 | 8.5 | 3.2 |
| **Methods applied at end of experiment** |  |  |  |  |  |  |
| SEM (n) | N/A | N/A | 4 | 8 | 4 | N/A |
| FISH (n) | 4 | 6 | 6 | 5 | 5 | N/A |

**Supplementary Table S5. The symbiotic and aposymbiotic *Z. niveum* phenotypes**

Data of symbiotic and aposymbiotic morphs grown in the same chamber (number 68) are presented as median and interquartile range of estimated number of branches and survival on day 7, and estimated life span, estimated number of branches at the estimated time of vertex. Fitness drop measured as relative reduction in number of branches between symbiotic and aposymbiotic colonies.

|  | Experiment 68  Symbiotic morphs | Experiment 68  Aposymbiotic morphs |
| --- | --- | --- |
| Number of branches | 22  (18, 29)  n=21 | 6  (5, 6)  n=21 |
| Estimated number of zooids | 107 | 24 |
| Survival (%) | 66  n=32 | 100  n=46 |
| Estimated lifespan  (days) | - | 13.2 |
| Estimated max. number of branches | - | 5.8 |
| Estimated time  of vertex (day) | - | 6.4 |

Fitness 100 27

(relative growth reduction)
